# SARS‐CoV‐2 infections in households in a peri‐urban community of Lima, Peru: A prospective cohort study

**DOI:** 10.1111/irv.12952

**Published:** 2021-12-27

**Authors:** Claudio F. Lanata, Ana I. Gil, Lucie Ecker, Rubelio Cornejo, Stefano Rios, Mayra Ochoa, Bia Peña, Omar Flores, Leigh M. Howard, Carlos G. Grijalva

**Affiliations:** ^1^ Instituto de Investigación Nutricional Lima Peru; ^2^ Vanderbilt University Nashville Tennessee USA; ^3^ London School of Hygiene and Tropical Medicine London UK; ^4^ Vanderbilt University and Vanderbilt University Medical Center Nashville Tennessee USA

**Keywords:** cohort, COVID‐19, incidence, Peru, SARS‐CoV‐2

## Abstract

**Background:**

We assessed the prevalence and incidence of SARS‐CoV‐2 infections in a prospective study of households in Lima, Peru.

**Methods:**

Households with a child, a young adult 18–50 years, and an adult age >50 years in peri‐urban Lima were followed with twice‐a‐week household visits during a 2‐month period. Nasal swabs and saliva specimens were collected twice weekly, and nasopharyngeal swabs were collected weekly from each participant, regardless of symptoms. Laboratory‐confirmed SARS‐CoV‐2 infection was defined by two RT‐PCR tests from any of the collected specimens within a week. Blood samples collected at enrollment and end of follow‐up were tested with rapid serological tests. We calculated the prevalence and incidence of laboratory‐confirmed SARS‐CoV‐2 infections.

**Results:**

We enrolled 132 participants from 44 households: 44 children, 44 young adults, and 44 older adults. A total of 13 SARS‐CoV‐2 infections were detected in eight households, for an overall period prevalence of 9.85% (95% confidence interval [CI]: 5.35–16.25). Most (61.54%) infections were symptomatic. Eight of 11 (72.73%) SARS‐CoV‐2 detections corresponded to the Lambda variant. During 218.79 person‐months at risk of follow‐up, there were six new SARS‐CoV‐2 infections detected (2.74 per 100 person‐month, 95% CI: 1.25–6.04). At enrollment, 59 of 128 participants tested had positive SARS‐CoV‐2 IgG serology (46.09%, 95% CI: 37.25–55.12). Five of six new infections occurred among participants with negative baseline serology.

**Conclusions:**

We demonstrated high incidence of SARS‐CoV‐2 infections in households, especially among subjects without evidence of prior infection, most of them not detected by the Ministry of Health system.

## 1 INTRODUCTION

The ongoing SARS‐CoV‐2 pandemic has disproportionally affected individuals with limited access to diagnostic and healthcare services. Yet, most available estimates of disease incidence come from populations with good access to those services, primarily high‐income countries.^1^, ^2^, ^3^, ^4^ Moreover, a majority of incidence estimates are based on passive reporting of laboratory results from testing centers or healthcare locations.^4^, ^5^ Reliable estimates of incidence from the community are limited, and data from low‐ to middle‐income countries (LMIC) are lacking.

Households, where individuals share common areas and usually interact in close proximity, provide an optimal venue for viral transmission. Whereas several prior studies have examined household transmission of influenza and other respiratory viruses,^6^ few studies have examined the incidence of SARS‐CoV‐2 infections in households.^1^, ^4^, ^7^

We conducted a prospective cohort study in households from a densely populated peri‐urban area in Lima, Peru. We sought to investigate the frequency of SARS‐CoV‐2 infections, independent of respiratory symptoms, and explore the patterns of SARS‐CoV‐2 infections in households.

## 2 METHODS

This was a prospective cohort study of households conducted from December 9, 2020, through March 19, 2021. Enrolled participants were followed through twice‐a‐week household visits, with systematic data collection on respiratory symptoms and healthcare use. Respiratory specimens were systematically collected during household visits, regardless of the presence of symptoms, for laboratory detection of viral infections. Baseline and follow‐up blood samples were obtained at home to determine serologic evidence of prior SARS‐CoV‐2 infections. No COVID‐19 vaccines were available to the study population during the study period.

### 2.1 Study population and selection criteria

Households were eligible for participation if they included at least three consenting members—one person <18 years, one 18–50 years, and one >50 years old—who were available during weekday working hours to be visited at home or in a nearby working area and had no plans to moving out of the area within the planned study follow‐up period. Additional household members could not be enrolled due to budget constraints; sociodemographic information of these was obtained from consenting members; however, samples and follow‐up information were obtained only from the three consenting members.

### 2.2 Enrollment and follow‐up

Identification of eligible households was performed through a house‐to‐house screening census within the study area in the San Juan de Lurigancho District of Lima. Households identified as eligible were approached for enrollment.

### 2.3 Ethical approvals

The study was approved by the ethics review boards (ERBs) of the *Instituto de Investigacion Nutricional* and Vanderbilt University. All adults signed their own ERB‐approved written informed consent form (ICF). For individuals under 18 years of age, either the mother or the father signed an ICF. Additionally, children 8 years and older signed a written informed assent form. All study participants were encouraged to use their regular health providers for any illness suspected to be related to COVID‐19, including their own diagnostic methods and treatment protocols. Laboratory tests were conducted after the surveillance was terminated to maintain blinding of participants and study members on viral infection status. After study completion, a final home visit was done to inform study participants about the study results and their own samples' results.

### 2.4 Collection of study specimens

During follow‐up household visits, trained fieldworkers collected twice‐weekly nasal swabs and weekly nasopharyngeal (NP) swab specimens. Nasal swabs were collected using Polyester Swabs (Puritan®) in viral transport media (VTM Remel®). NPs were collected using rayon swabs in STGG media. After collection, samples were maintained in cold boxes and transported within 4 h of collection to the field laboratory. Study participants were trained to self‐collect saliva samples on the same days nasal swabs were taken. For saliva sample collections, study participants were given a non‐sterile capped plastic container and instructed to fill it with about 5 ml of saliva passively collected either before breakfast or at least 3 h after eating, close the container tightly, and keep the samples in refrigerators (if available) until samples were picked up by fieldworkers.

In addition, we obtained venous blood samples (3 ml) from participants at enrollment and at the end of the 2‐month surveillance period. Blood samples were transported at room temperature to the field laboratory where serum was separated, and two aliquots were initially frozen at −20°C. At the end of each week, blood samples were transported to the central laboratory for long‐term storage at −80°C.

### 2.5 Laboratory procedures

The saliva and nasal swab specimens were distributed in a maximum of four aliquots in cryovials as soon as they were received by the laboratory. Each aliquot was frozen at −80°C for long‐term storage. To increase the efficiency of RT‐PCR testing, we created separate pools of nasal and saliva samples combining sample aliquots (100 μl) collected on the same week from all members of the same household (pools sizes ranged from 2 to 12 samples).^8^ NP samples were tested separately and on an individual basis (i.e., without pooling). All respiratory and saliva samples were tested for SARS‐CoV‐2 using RT‐PCR. If a pool tested positive, each individual sample from the pool participants was then tested. For all specimens, RNA extraction was done using the MagMAX^R^ Viral/Pathogen Nucleic Acid Isolation Kit by Thermo Scientific, utilizing the KingFisher DUO^R^ prime extractor, using 200 μl of sample that generated 50 μl volume of extracted RNA. For the real‐time RT‐PCR, we used the TaqPath^R^ COVID‐19 CE‐IVD RT‐PCR Kit utilizing the QuantStudio5^R^ thermocycler. Positive samples with a CT value of 33 or lower were sent to the Genomic Laboratory of the Peruvian University Cayetano Heredia for viral sequencing.^9^

From blood samples, IgM and IgG antibodies against the N (nucleocapsid) protein of the SARS‐CoV‐2 were measured using sera aliquots in the SD Biosensor Standard Q COVID‐19 IgM/IgG Combo^R^, a rapid serology test that was used by the Peruvian Ministry of Health to diagnose COVID‐19 cases during most of 2020. Seropositivity was defined as a positive IgG result. Seroconversion was defined as the shift from seronegative IgG blood sample at baseline to a seropositive IgG blood sample at the end of surveillance. IgM positivity was not considered for these assessments.

### 2.6 Infection episodes and symptoms data

SARS‐CoV‐2 infections were identified through RT‐PCR. To increase the specificity of our viral infection determinations, we defined a SARS‐CoV‐2 infection with two or more positive test results from any respiratory specimen type on the same day or within 7 days of each other. We defined a new infection episode as those that started without a positive test result within the prior 7 days. Each day of follow‐up was characterized according to the reported presence of respiratory symptoms. We defined a symptomatic SARS‐CoV‐2 infection episode using a recommended Center for Disease Control and Prevention‐CDC's operational definition,^10^, ^11^ that is, when participants reported at least 1 day with: cough, difficulty breathing, or loss of smell or flavors, or the combination of two or more of fever (reported or measured), throat sore, nausea or vomiting, diarrhea, or nasal congestion during the episode. Infection episodes without any of the aforementioned COVID‐like symptoms were defined as asymptomatic.

### 2.7 Data analysis

In‐house data entry and management software were used to enter data into a study database, with internal (within study forms) and external (between study forms) data quality checks. For prevalence estimates, we used infections detected at any time of the study period. Participants with positive samples at enrollment were included in the prevalence estimate but were not considered for incidence calculation (i.e., were not at risk). Because the follow‐up period was short, we considered that a participant was no longer at risk once infected and did not attempt to assess recurrent infections. Incidence rates were first calculated considering all person‐days observed during the surveillance period. We also estimated incidence rates selecting all person‐days observed prior to the start of a new infection and including the day when the new infection definition was met, as period at risk. All person‐days observed after an initial infection were considered not at risk. We then applied the incidence (person‐time at risk) estimated to the census population of the sectors of Metropolitan Lima classified as peri‐urban to estimate the number of SARS‐CoV‐2 infections per day and compare that number with the daily number of COVID‐19 cases reported by the Peruvian Ministry of Health for that sector of Lima, as well as the daily number of COVID‐19 attributable deaths reported by the national system of death certificates.^12^

## 3 RESULTS

### 3.1 Enrollment

**FIGURE 1:**
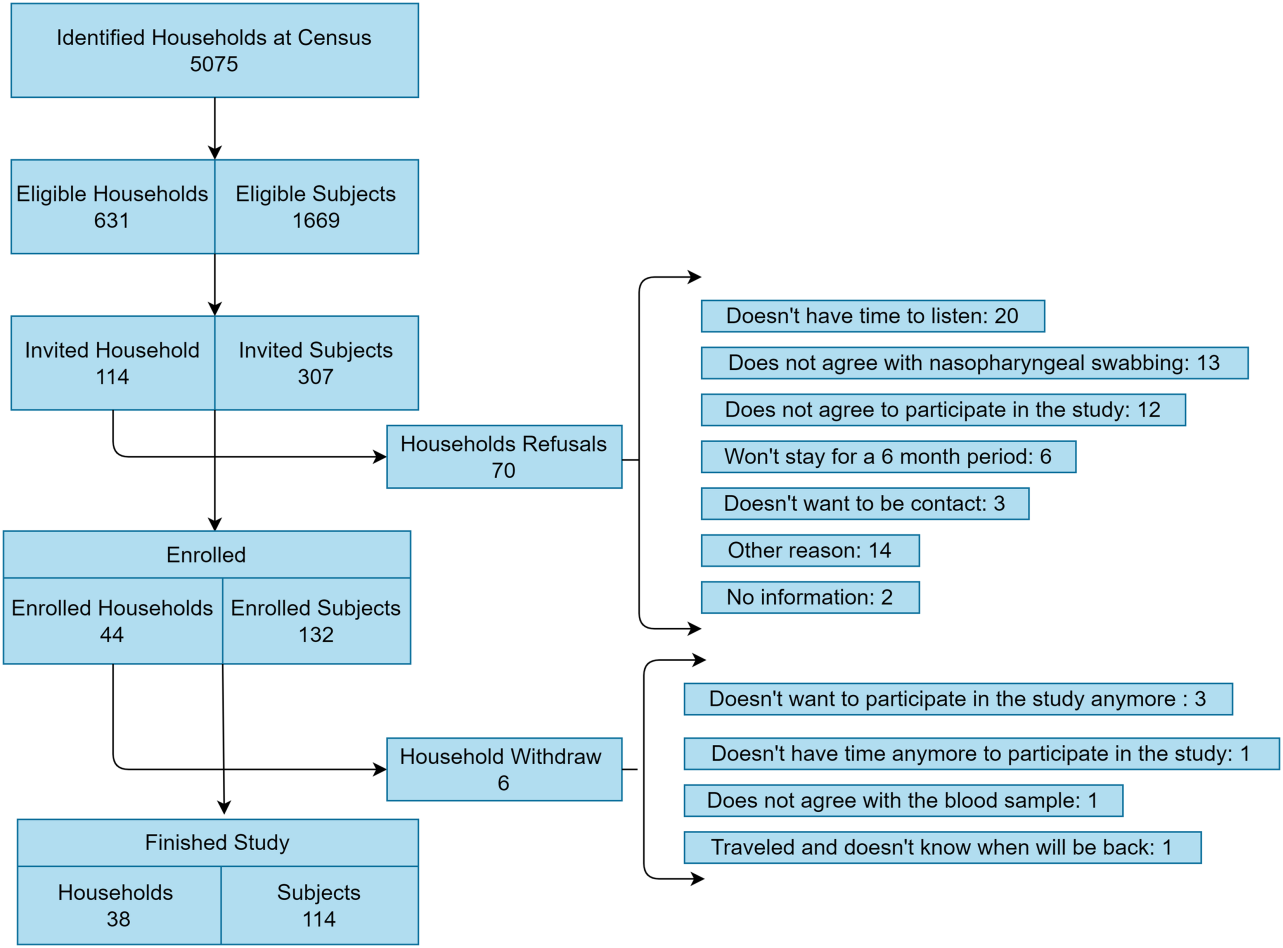
Flow diagram of the 44 households enrolled into the study, peri‐urban Lima, Peru. Dec 2020 to Jan 2021

In the screening census of the study area, 631 potential eligible households were initially identified. After approaching 114 eligible households by trained fieldworkers, 44 households were enrolled in the study, with one individual for each study age group providing written consent/assent (Figure 1).

### 3.2 Study population

**FIGURE 2:**
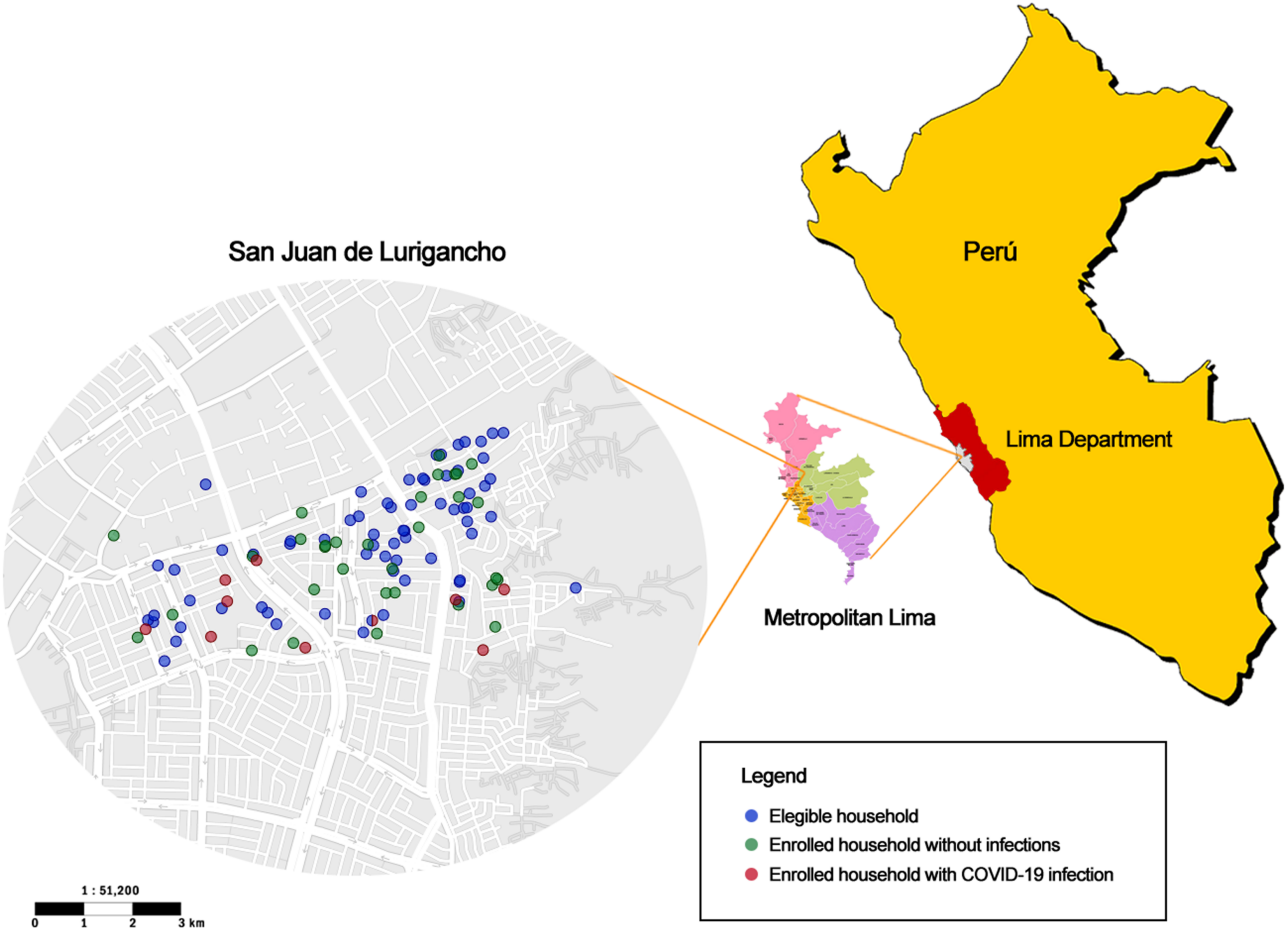
Location of eligible and enrolled households and location of study area in the San Juan de Lurigancho District, Lima, Peru, Dec 2020 to Mar 2021

A total of 132 subjects from 44 households were enrolled in the study. Figure 2 shows the GPS location of the 44 households enrolled, which were homogeneously distributed within the study area and among those eligible households. The mean age of the 44 study participants <18 years old was 3.66 years ± 2.50, range 1–17 (59.09% female); for the 44 participants 18–50 years old, the mean age was 32.93 years ± 7.08, range 19–49 (95.45% female); and for the 44 participants >50 years old, the mean age was 63.75 years ± 6.79, range 51–85 (75.00% female). The sociodemographic characteristics of the 40 enrolled households that completed the socioeconomic survey are described in Table 1. Most basic services (e.g., water and sewage) were available in enrolled households. Few enrolled members were students, due to school closures due to the pandemic and being at summertime when schools are closed. Groceries purchasing were done either daily or one to three times per week in the local food market, and most household members utilized different types of public transportation. From the 44 households initially enrolled, 38 completed follow‐ups, encompassing a total of 114 household members for an 86.36% retention through the end of the study (Figure 1).

**TABLE 1:** Characteristics of the 40 households (Panel A) and its 283 members (Panel B) participating in the cohort study, peri‐urban Lima, Peru, Dec 2020 to Jan 2021

A	
Households characteristics	Result
Number of bedrooms per household	3.67 ± 1.42 (1–8)^a^
Household materials and selected goods	
Walls with bricks/cement	87.50%
Piped potable water at home	100.00%
Sewage connection at home	100.00%
Electricity supply	100.00%
Gas kitchen stove	65.00%
Computer	60.00%
Microwave oven	47.50%
Refrigerator	92.50%
Television set	100.00%
Private car ownership	32.50%
Frequency of grocery purchasing	
Daily	30.00%
1–3 times per week	67.50%
Every 15 days	2.50%
Used public transportation	
Bus	42.50%
Microbus	7.50%
Shared private cars	5.00%
Taxis	2.50%
Motorcycle taxis	37.50%
Lima Metro	5.0%
B	
Characteristic of household members	Result
Number of household members	
0–17 years	2.38 ± 1.05(1–5)^b^
18–50 years	3.10 ± 1.30(1–7)^b^
>50 years	1.60 ± 0.63(1–3)^b^
Total per household	7.08 ± 2.00 (4–11)^b^
Percentage female	57.60%
Educational level achieved by household members	
Infants/Pre‐kinder	19.08%
Primary education	14.49%
Secondary education	29.33%
Technical/university	34.63%
Illiterate^c^	2.47%
Main activity of household members	
Stay at home	61.80%
Self‐employed	14.84%
Work on a payroll job	13.43%
Sales or local commercial activity	4.90%
Student	2.50%
Retired	1.40%
Unknown	1.13%
Health coverage of household members	
Ministry of Health system	59.36%
Social Health Care System	32.50%
Private Health Care system	1.40%
Armed Forces Health Care System	1.10%
No health coverage	4.20%
Unknown	1.44%

^a^ Mean ± sd (min − max).

^b^ Mean (min − max).

^c^ Illiterate applies to people older than 15 years old.

### 3.3 Serological evidence of prior infection

We obtained a blood sample from 128 study members at baseline and 111 at end of the surveillance period. A baseline positive IgG test was documented for 46.09% of participants (95% CI: 37.25–55.12), without significant differences across age groups. At the end of surveillance, the IgG positivity was 53.15% (95% CI: 43.45–62.69). Among the 110 participants with paired samples available, IgG seroconversion was observed in 11 individuals (10.00%, 95% CI: 5.10–17.19) during the study period. All but one seroconversions occurred in participants that had at least one positive PCR for SARS‐CoV‐2 during the study period.

### 3.4 Prevalence and incidence of SARS‐CoV‐2 infections

There were 13 SARS‐CoV‐2 infections detected among the 132 persons observed (9.85% period prevalence, 95% CI: 5.35–16.25) (Table 2). Eleven (84.6%) of these detections were sequenced, and 8 (72.72%) were determined to be the SARS‐CoV‐2 C‐37, Lambda variant, the other three being from the B.1.1.348 PANGO_lineage. Fifty percent of infections in the <18‐year age group, 67% in the 18–50 years old, and 100% of infections in the >50 years old were symptomatic. An infected study participant was admitted to a private clinic and treated with low‐flow oxygen. An additional infected study participant consulted an outpatient service and was sent home without oxygen treatment.

**TABLE 2:** Prevalence and incidence of SARS‐CoV‐2 infections in 44 households, peri‐urban Lima, Peru, Dec 2020 to Mar 2021

	Period prevalence			Incidence		
Age group (years)	N	Number of infections	Prevalence % (95% CI)	Number of persons‐mo at risk observed	Number of new infections	Rate: Number of new infect/100 persons‐mo at risk (95% CI)
0–17	44	4	9.09 (2.53–21.67)	72.02	1	1.39 (0.20–9.72)
18–50	44	6	13.64 (5.17–27.35)	72.07	3	4.16 (1.37–12.60)
>50	44	3	6.98 (1.43–18.66)	74.71	2	2.68 (0.68–10.51)
Total	132	13	9.85 (5.35–16.25)	218.79	6	2.74 (1.25–6.04)

**FIGURE 3:**
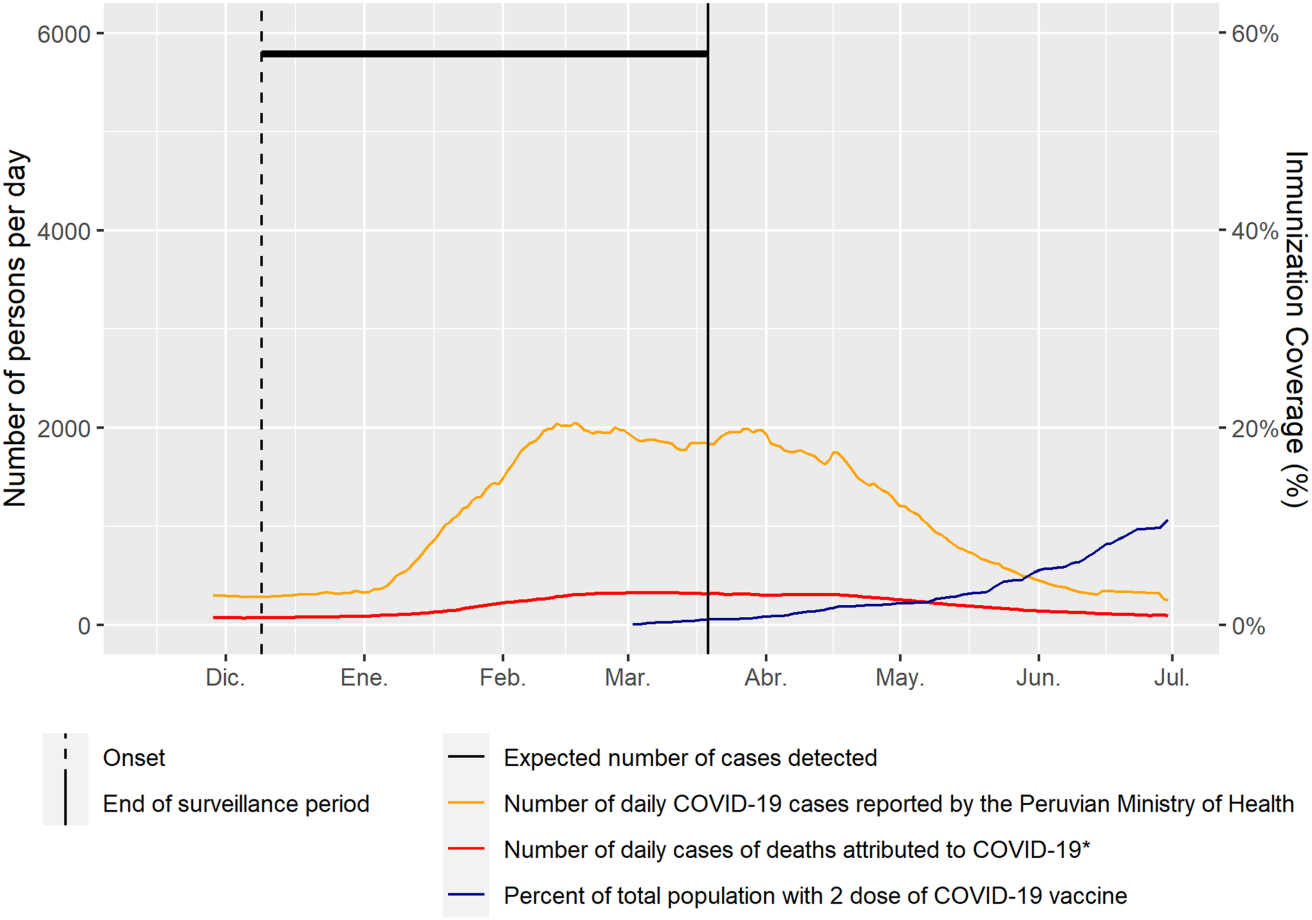
Number of estimated daily COVID‐19 cases, daily COVID‐19 cases reported by the Peruvian Ministry of Health, daily of excess of total deaths estimated by the Peruvian SINADEF system, and COVID‐19 immunization coverage in peri‐urban districts of Lima, Peru. Dec 2020 to Jul 2021

Six infections were considered new infections, with an incidence rate of 2.56 episodes/100 person‐months observed, with point estimates of 1.28 (95% CI: 0.18–8.95), 3.84 (95% CI: 1.26–11.63), and 2.57 (95% CI: 0.66–10.11) episodes/100 person‐months observed for children, young adults, and older adults, respectively. These rates were slightly higher when they were expressed per 100 person‐months at risk (Table 2). When we applied the rate of 2.74 new infections per 100 person‐months at risk to the total peri‐urban population of Metropolitan Lima and estimated the number of cases per day occurring in that sector of Lima, we have estimated that only about a third of infections have been detected by the official reporting system (Figure 3).

Five of the six new infections occurred in individuals who were IgG seronegative at baseline, and one new infection in a seropositive asymptomatic participant. The incidence rate among IgG seronegative individuals was 4.41 (95% CI: 1.87–10.39) episodes/100 person‐months at risk (Table 3). Four of these new infections had a demonstrated seroconversion; and one case had no blood sample available at the end of the follow‐up period. Another individual had IgG seroconversion without an infection detected in respiratory samples and remained asymptomatic during the 2‐month follow‐up period.

**TABLE 3:** Incidence of SARS‐CoV‐2 infections in households, peri‐urban Lima, Peru, Dec 2020 to Mar 2021, according to serological evidence of prior infection at enrollment

	Incidence					
	Without serological evidence of infection at enrollment^a^			With serological evidence of infection at enrollment^b^		
Age group (years)	Number of persons/mo at risk	Number of new infections	Rate: number of new infect/100 person‐mo at risk (95% CI)	Number of persons‐mo at risk observed	Numberof new infections	Rate: number of new infect/100 persons‐mo at risk (95% CI)
0–17	30.59	1	3.27 (0.48–22.47)	37.03	0	0 (NC)
18–50	39.16	2	5.11 (1.32–19.70)	31.87	1	3.14 (0.46–21.60)
>50	43.66	2	4.58 (1.18–17.74)	31.05	0	0 (NC)
Total	113·41	5	4.41 (1.87–10.39)	99.94	1	1.00 (0.14–7.03)

Abbreviation: NC, not calculated.

^a^ IgG negative rapid test in serum.

^b^ IgG positive rapid test in serum.

## 4 DISCUSSION

During 2 months of intensive household‐based prospective surveillance, the prevalence of SARS‐CoV‐2 infections was 9.85%, and the incidence was 2.74 infections per 100 person‐months at risk. Nearly half (46.09%) of enrolled individuals were IgG seropositive at baseline, and an additional 10% of individuals seroconverted during the 2‐month period of intensive surveillance. Almost all new infections were observed among individuals who were seronegative at baseline.

To our knowledge, this is the first prospective cohort study for SARS‐CoV‐2 surveillance conducted in households in a densely populated area of a Latin American LMIC. This longitudinal study maintained intensive follow‐up through twice‐weekly household visits with collection of nasal swabs and saliva samples and a weekly NP swab collection, regardless of symptoms. The study was conducted at the time of the peak of the second wave of the SARS‐CoV‐2 epidemic affecting Lima, associated with the Lambda^13^ virus variant (Figure 3). Our data suggest that individuals 18–50 years of age may have higher rates of infection and may play an important role in the transmission of the virus. In contrast, children <18 years old were less frequently infected, suggesting a different role in household transmission. At the time of the study, Peru national recommendations requested that all persons considered at high risk for severe infection to stay at home and the rest of the population should use masks and avoid large gatherings and close contact with others. In our study population, household members had frequent exposures as they left their houses to purchase groceries daily or one to three times per week at crowded local markets and frequently used public transportation systems.

A recently published cohort study of 1236 participants in a convenience sample of 310 households from New York City and selected counties in Utah, USA, reported incidence estimates among participants without a known history of prior SARS‐CoV‐2 infection.^7^ Follow‐up included weekly surveillance and infections were ascertained by self‐administered nasal swabs for SARS‐CoV‐2 detection, complemented by additional nasal swabs and saliva samples when participants developed symptoms compatible with COVID‐19. The age‐adjusted incidence rate of SARS‐CoV‐2 infections detected by RT‐PCR was 5.8 (95% CI, 3.1–8.4) per 1000 person‐weeks at risk, which is equivalent to 2.52 infections per 100 person‐months, like the overall incidence rate observed in our study.^7^ A higher incidence rate (3.34 infections per 100 person‐months) was reported for New York City than for Utah (1.65 infections per 100 person‐months). Nevertheless, after exclusion of those individuals with serological evidence of prior SARS‐CoV‐2 infection, our study demonstrated an incidence rate of 4.41 infections per 100 person‐months at risk, which is higher than the rate reported in the US study. Of note, COVID‐19 vaccines were introduced during their study period in the United States, from September 2020 through April 2021, and no SARS‐CoV‐2 infections were detected among vaccinated individuals, which could explain in part their lower incidence rates.

Interestingly, only one (8%) of the 13 infections detected in our study was detected by the Ministry of Health system. In addition, as shown in Figure 3, we have estimated that only about a third of infections have been detected by the official reporting system in Lima. This implies that pandemic control efforts concentrated only on cases (and contacts) detected through passive surveillance systems that rely on encounters with the healthcare system may underestimate viral activity as demonstrated in this densely populated peri‐urban area of an LMIC. As access and use of healthcare services is typically limited in these settings, and more so during the pandemic, our observations suggest that the observed underestimation could be a widespread issue. Without adoption of proactive mass screening using highly sensitive molecular techniques for symptomatic and asymptomatic individuals in areas of high transmission, control measures will continue to have limited success in containing this pandemic. It is important to note that rapid identification of first infections should be paired with effective isolation of infected individuals and quarantine of close contacts.

This study has several limitations. Due to limited availability of research support, our study focused on a limited number of households and had a short follow‐up period. Because of participants' availability and participation preferences, our study sample had an overrepresentation of females. This study focused on only three household members from each participating household. The incidence of infections among other members living in the same households with our participants was not observable. The mean number of household members in enrolled households (7.08 ± 2.0) was higher than the 4.4 ± 2.2 (range 3–5) members per household observed in a previous census of households in the same study area with the only requirement to have a child <5 years, so this study represents households with larger family size. The study is based on a convenience sample of households that agreed to participate in the study, and findings from this assessment may not be directly generalizable to other settings. Though we observed lower incidence rates of infections among children in this study, it is important to underscore that during the study period, schools in Peru were closed, which may have limited their exposure to the virus. Nevertheless, besides age group requirements, the study did not have major selection criteria in an attempt to increase generalizability. Of note, the San Juan de Lurigancho District had the highest number of reported COVID‐19 cases and deaths in Lima throughout the pandemic. The specific factors driving this high incidence of reported cases are currently unknown. It would be informative to conduct similar studies in other peri‐urban populations in LMIC.

The study has several strengths. The cohort selection criteria did not focus on a particular high‐risk group, like health workers or persons under a particular insurance coverage, that may not represent the general population. The intensity of surveillance, and most importantly, the systematic sampling with twice‐a‐week visits with collection of nasal and saliva samples, and weekly NPs conducted throughout the surveillance period, allows high sensitivity to detect any SARS‐CoV‐2 infection regardless of presence of symptoms. The combination of molecular testing with serology has also provided important information. Most new infections occurred among those who were IgG seronegative at baseline, suggesting the protective effect of prior infections, as demonstrated elsewhere.^14^ Of note, no study participant received any COVID‐19 vaccine before or during the study. The prevalence of IgG seropositivity in study participants of 46.09% was higher than the rate of 20.8 (95% CI 17.2–23.5) of individuals with either IgM or IgG seropositivity in a cross‐sectional serosurvey done in Lima at mid‐2020,^15^ suggesting a high intensity of prior infections in the study area.

During this study, Peru started introducing the Sinopharm vaccine to the healthcare providers working in the first line of pandemic control, but no vaccination occurred in our study population. As shown in Figure 3, the proportion of peri‐urban population of Lima being given two doses of a COVID‐19 vaccine started to increase at the end of our study period.

In summary, we demonstrated high rates of infection in a highly populated peri‐urban area of Lima, Peru. Young adults were commonly infected and appear to bring the infections into their households. The great majority of documented infections occurred among individuals without evidence of prior infection, and only a small fraction of all detected infections interacted with the healthcare system and were ultimately identified by the regular surveillance system of the Peruvian Ministry of Health. Widespread efforts for timely identification of symptomatic and asymptomatic infected individuals in the community may be an important consideration for successful control programs. Similar longitudinal studies with systematic testing could help characterize the activity of other viral variants and incidence of infections in other populations.

## AUTHOR CONTRIBUTIONS

**Claudio Lanata:** Conceptualization; data curation; formal analysis; funding acquisition; investigation; methodology; project administration; resources; supervision; validation; visualization. **Ana Gil:** Conceptualization; data curation; formal analysis; investigation; methodology; project administration; resources; supervision; validation; visualization. **Lucie Ecker:** Conceptualization; data curation; formal analysis; investigation; methodology; project administration; resources; supervision; validation; visualization. **Rubelio Cornejo:** Conceptualization; data curation; formal analysis; investigation; methodology; resources; software; supervision; validation; visualization. **Stefano Rios:** Data curation; formal analysis; methodology; software; supervision; validation; visualization. **Mayra Ochoa:** Data curation; formal analysis; investigation; methodology; resources; supervision; validation; visualization. **Bia Peña:** Data curation; formal analysis; investigation; methodology; supervision; validation; visualization. **Omar Flores:** Data curation; formal analysis; investigation; methodology; resources; supervision; validation; visualization. **Leigh Howard:** Conceptualization; formal analysis; investigation; methodology; validation; visualization. **Carlos Grijalva:** Conceptualization; formal analysis; funding acquisition; investigation; methodology; resources; supervision; validation; visualization.

## CONFLICT OF INTERESTS

Funds were provided to the Institution, not to the authors. CFL is a member of WHO COVID‐19 vaccine effectiveness working group and WHO Product Development Advisory Group. CGG reports consultancy fees from Pfizer, Merck, and Sanofi‐Pasteur and grants from Campbell Alliance/Syneos Health, CDC, NIH, the Food and Drug Administration, AHQR, and Sanofi, outside the submitted work. LMH reports grant funding from the Infectious Disease Society of America Education and Research Fund supported by Pfizer and from NIH, outside the submitted work.

## ETHICS STATEMENT

The study was approved by the ethics review boards (ERBs) of the *Instituto de Investigacion Nutricional* and Vanderbilt University. All participants (or one parent for participants <18 years of age) signed an ERB‐approved written informed consent form. Children 8–17 years of age signed their own assent form.

### PEER REVIEW

The peer review history for this article is available at https://publons.com/publon/10.1111/irv.12952.

## Data Availability

Data without any participant's identifying information, could be provided after all final analysis are completed, if requested to the correspondence author. After approval, and the signature of a data transfer agreement form, data could only be used for the purpose requested, unless a new request is made.
